# Supplementary material for: Prevalence and the association between clinical factors and Diabetes-Related Distress (DRD) with poor glycemic control in patients with type 2 diabetes: A Northern Thai cross-sectional study
Source: PLoS One. 2023 Nov 27;18(11):e0294810. doi: 10.1371/journal.pone.0294810 (PMC10681199; doi:10.1371/journal.pone.0294810)
Supplement: S1 File — (DOCX) [file pone.0294810.s001.docx]

**S1. Study flow**

All type 2 diabetic patients who visited the clinic and met the inclusion criteria were voluntarily invited to participate by the outpatient clinic's nurse (the first nurse).

The data were sent to the main investigator.

Only dietary assessment data (24-hour dietary recall) were sent to a dietitian for evaluating total calorie and carbohydrate intake.

Another research assistant (the second research assistant physician) was assigned to double-check the data for completeness, consistency, reliability, and to prevent transcription errors.

Afterward, a research data collection assistant (the first research assistant physician), retrieved data including weight, height, waist circumference, blood pressure, FBS, HbA1c, and diabetes treatment modalities from the electronic medical records of that day.

The same registered nurse (the second nurse) conducted questionnaire interviews and performed assessments using standard tools, including comorbidities, duration of type 2 diabetes, smoking, alcohol consumption, cognitive screening (Mini-Cog), self-care behaviors (self-care behavior questionnaire), physical activity (GPAQ), dietary assessments (24-hour dietary recall), sleep quality (PSQI), depression screening (9Q),

and DRD assessment (DDS-17).

Demographic data were collected through a self-administered paper-based questionnaire, which included information such as age, sex, occupation, personal monthly income, education, marital status, and health insurance. The information that patients had completed was then submitted to the same registered nurse (the second nurse) for a thorough check to ensure completeness.

Participants who were willing to participate in the study were directed to a registered nurse (the second nurse) who served as a research data collector and was trained to conduct interviews. The registered nurse provided information about the data collection process and obtained written informed consent from the patients.

Patients would continue their routine examination and treatment with the OPD family physicians. Afterwards, they would be discharged and scheduled for routine follow-up appointments.
