# Supplementary material for: Prevalence and the association between clinical factors and Diabetes-Related Distress (DRD) with poor glycemic control in patients with type 2 diabetes: A Northern Thai cross-sectional study
Source: PLoS One. 2023 Nov 27;18(11):e0294810. doi: 10.1371/journal.pone.0294810 (PMC10681199; doi:10.1371/journal.pone.0294810)
Supplement: Attachment — Submitted filename: PONE-D-23-07649_reviewer comments.pdf [file pone.0294810.s002.pdf]

PLOS ONE

Physical, behavioral, and psychosocial factors associated with uncontrolled type 2 diabetes: a Northern Thai cross-sectional study.

--Manuscript Draft--

**Manuscript Number:**	PONE-D-23-07649
**Article Type:**	Research Article
**Full Title:**	Physical, behavioral, and psychosocial factors associated with uncontrolled type 2 diabetes: a Northern Thai cross-sectional study.
**Short Title:**	Physical, behavioral, and psychosocial factors associated with uncontrolled type 2 diabetes.
**Corresponding Author:**	nalinee yingchankul<br>Chiang Mai University Faculty of Medicine<br>Muang, THAILAND
**Keywords:**	uncontrolled diabetes; diabetes related distress; factor; behavior; psychosocial; DDS
**Abstract:**	Enclosed please find an original research article to be considered for publication in the PLOS ONE Journal, entitled “Physical, behavioral, and psychosocial factors associated with uncontrolled type 2 diabetes: a Northern Thai cross-sectional study”. We confirm that this manuscript has not been published elsewhere and is not under consideration by any other journals. There is no prior interaction with PLOS regarding the submitted manuscript. All the authors have approved the manuscript and agree with its submission to the journal. We believe that our paper would be of interest to the readers of journal.<br>Background<br>Glycemic control is important to prevent diabetic complications. However, there is a lack of studies regarding psycho-social factors especially diabetes-related distress (DRD) in Thailand. This study aimed to investigate the prevalence and associated factors of uncontrolled type 2 diabetes categorized in physical, behavioral, and psycho-social dimensions.<br>Methods<br>A prospective cross-sectional study was conducted on 127 type 2 diabetic patients between December 2021 and March 2022 at Maharaj Nakorn Chiang Mai Hospital, Thailand. Data collection included physical data (clinical demographic, medical, and diabetic information), behavioral data (self-care behavior, physical activity, dietary assessment, smoking, alcohol consumption, and sleep quality), and psycho-social data (depression and DRD). The target HbA1C was determined based on The American Diabetes Association (ADA) Guideline 2021. Univariable associations between potential factors and uncontrolled type 2 diabetes were evaluated using independent t-test and Fisher’s exact test. Factors that showed statistically significant results in the univariable analysis (p-value<0.05) were further explored using multivariable logistic regression analysis.<br>Results<br>The prevalence of uncontrolled type 2 diabetes was 29.1%. Multivariable analysis revealed that DRD (OR 14.20, 95% CI 3.76-53.64, p<0.001), age under 65 years old (OR 6.40, 95% CI 2.07-19.77, p=0.001), and obesity (BMI ≥ 25) (OR 2.96, 95% CI 1.05-8.39, p=0.041) were significantly associated with uncontrolled type 2 diabetes. Three dimensions of DRD were associated with uncontrolled type 2 diabetes, including emotional distress (OR 4.23, 95% CI 1.51-11.85, p=0.006), regimen-related distress (OR 6.00, 95% CI 1.88-19.18, p=0.003), and interpersonal distress (OR 5.25, 95% CI 1.39-20.02, p=0.015).<br>Conclusion and recommendation<br>Age, obesity, and DRD are associated with uncontrolled type 2 diabetes. Highlights the need for a holistic approach that considers both physical and psycho-social factors to prevent diabetic complications. Further studies with broader populations using a cohort-based design are recommended.
**Order of Authors:**	Achiraya Ruangchaisiwawet
	Nalinee Yingchankul

Powered by Editorial Manager® and ProduXion Manager® from Aries Systems Corporation

	Narumit Bankhum
	Krittai Tanasombatkul
	Phichayut Phinyo
**Additional Information:**	
**Question**	**Response**
**Financial Disclosure**<br><br>Enter a financial disclosure statement that describes the sources of funding for the work included in this submission. Review the submission guidelines for detailed requirements. View published research articles from *PLOS ONE* for specific examples.<br><br>This statement is required for submission and **will appear in the published article** if the submission is accepted. Please make sure it is accurate.<br><br>**Unfunded studies**<br>Enter: *The author(s) received no specific funding for this work.*<br><br>**Funded studies**<br>Enter a statement with the following details:<br>• Initials of the authors who received each award<br>• Grant numbers awarded to each author<br>• The full name of each funder<br>• URL of each funder website<br>• Did the sponsors or funders play any role in the study design, data collection and analysis, decision to publish, or preparation of the manuscript?<br>• **NO** - Include this sentence at the end of your statement: *The funders had no role in study design, data collection and analysis, decision to publish, or preparation of the manuscript.*<br>• **YES** - Specify the role(s) played.<br><br>* typeset	This study was supported by Faculty of Medicine, Chiang Mai University grant number 035/2566. https://w1.med.cmu.ac.th/research/Homepage/Default.html#. The funders had no role in study design, data collection and analysis, decision to publish, or preparation of the manuscript.
**Competing Interests**<br><br>Use the instructions below to enter a competing interest statement for this submission. On behalf of all authors, disclose any competing interests that could be perceived to bias this	The authors declare no conflict of interest.

work—acknowledging all financial support and any other relevant financial or non-financial competing interests.<br><br>This statement is required for submission and **will appear in the published article** if the submission is accepted. Please make sure it is accurate and that any funding sources listed in your Funding Information later in the submission form are also declared in your Financial Disclosure statement.<br><br>View published research articles from *PLOS ONE* for specific examples.<br><br>**NO authors have competing interests**<br><br>Enter: *The authors have declared that no competing interests exist.*<br><br>**Authors with competing interests**<br><br>Enter competing interest details beginning with this statement:<br><br>*I have read the journal's policy and the authors of this manuscript have the following competing interests: [insert competing interests here]*<br><br>* typeset	
**Ethics Statement**<br><br>Enter an ethics statement for this submission. This statement is required if the study involved:<br><br>• Human participants<br>• Human specimens or tissue<br>• Vertebrate animals or cephalopods<br>• Vertebrate embryos or tissues<br>• Field research<br><br>Write "N/A" if the submission does not require an ethics statement.	Ethical approval was obtained from the Research Ethics Committee, Faculty of Medicine, Chiang Mai University, Thailand (FAM-2564-08500). Written informed consent was obtained from the study participants after informing the purpose of the study.

Powered by Editorial Manager® and ProduXion Manager® from Aries Systems Corporation

General guidance is provided below. Consult the submission guidelines for detailed instructions. **Make sure that all information entered here is included in the Methods section of the manuscript.**<br><br>**Format for specific study types**<br><br>**Human Subject Research (involving human participants and/or tissue)**<br>• Give the name of the institutional review board or ethics committee that approved the study<br>• Include the approval number and/or a statement indicating approval of this research<br>• Indicate the form of consent obtained (written/oral) or the reason that consent was not obtained (e.g. the data were analyzed anonymously)<br><br>**Animal Research (involving vertebrate animals, embryos or tissues)**<br>• Provide the name of the Institutional Animal Care and Use Committee (IACUC) or other relevant ethics board that reviewed the study protocol, and indicate whether they approved this research or granted a formal waiver of ethical approval<br>• Include an approval number if one was obtained<br>• If the study involved *non-human primates*, add *additional details* about animal welfare and steps taken to ameliorate suffering<br>• If anesthesia, euthanasia, or any kind of animal sacrifice is part of the study, include briefly which substances and/or methods were applied<br><br>**Field Research**<br><br>Include the following details if this study involves the collection of plant, animal, or other materials from a natural setting:<br>• Field permit number<br>• Name of the institution or relevant body that granted permission	
**Data Availability**<br><br>Authors are required to make all data underlying the findings described fully	Yes - all data are fully available without restriction

available, without restriction, and from the time of publication. PLOS allows rare exceptions to address legal and ethical concerns. See the PLOS Data Policy and FAQ for detailed information.<br><br>A Data Availability Statement describing where the data can be found is required at submission. Your answers to this question constitute the Data Availability Statement and **will be published in the article**, if accepted.<br><br>**Important:** Stating 'data available on request from the author' is not sufficient. If your data are only available upon request, select 'No' for the first question and explain your exceptional situation in the text box.<br><br>Do the authors confirm that all data underlying the findings described in their manuscript are fully available without restriction?	
**Describe where the data may be found in full sentences. If you are copying our sample text, replace any instances of XXX with the appropriate details.**<br><br>• If the data are **held or will be held in a public repository**, include URLs, accession numbers or DOIs. If this information will only be available after acceptance, indicate this by ticking the box below. For example: *All XXX files are available from the XXX database (accession number(s) XXX, XXX.*).<br>• If the data are all contained **within the manuscript and/or Supporting Information files**, enter the following: *All relevant data are within the manuscript and its Supporting Information files.*<br>• If neither of these applies but you are able to provide **details of access elsewhere**, with or without limitations, please do so. For example:<br><br>*Data cannot be shared publicly because of [XXX]. Data are available from the XXX Institutional Data Access / Ethics*	All relevant data are within the manuscript and its Supporting Information files.

Committee (contact via XXX) for researchers who meet the criteria for access to confidential data.<br><br>*The data underlying the results presented in the study are available from (include the name of the third party and contact information or URL).*<br>• This text is appropriate if the data are owned by a third party and authors do not have permission to share the data.<br><br>* typeset	
Additional data availability information:	

Powered by Editorial Manager® and ProduXion Manager® from Aries Systems Corporation

Manuscript

Click here to access/download;Manuscript;Manuscript_DM_DDS_for_ploson

Physical, behavioral, and psychosocial factors associated with uncontrolled type 2 diabetes: a Northern Thai cross-sectional study.

Achiraya Ruangchaisiwawet[1], Narumit Bankhum[2], Krittai Tanasombatkul[1,3], Phichayut Phinyo[1,3,4], Nalinee Yingchankul[1]*

[1]Department of Family Medicine, Faculty of Medicine, Chiang Mai University, Chiang Mai, Thailand.
[2] Nutrition and Dietary service section, Faculty of Medicine, Chiang Mai University, Chiang Mai, Thailand.
[3] Center for Clinical Epidemiology and Clinical Statistics, Faculty of Medicine, Chiang Mai University, Chiang Mai, Thailand.
[4] Musculoskeletal Science and Translational Research (MSTR), Chiang Mai University, Chiang Mai, Thailand.

*Corresponding author
E-mail: nalineey@hotmail.com (NY)

Abstract

Background

Glycemic control is important to prevent diabetic complications. However, there is a lack of studies regarding psycho-social factors especially diabetes-related distress (DRD) in Thailand. This study aimed to investigate the prevalence and associated factors of uncontrolled type 2 diabetes categorized in physical, behavioral, and psycho-social dimensions.

Methods

A prospective cross-sectional study was conducted on 127 type 2 diabetic patients between December 2021 and March 2022 at Maharaj Nakorn Chiang Mai Hospital, Thailand. Data collection included physical data (clinical demographic, medical, and diabetic information), behavioral data (self-care behavior, physical activity, dietary assessment, smoking, alcohol consumption, and sleep quality), and psycho-social data (depression and DRD). The target HbA1C was determined based on The American Diabetes Association (ADA) Guideline 2021. Univariable associations between potential factors and uncontrolled type 2 diabetes were evaluated using independent t-test and Fisher's exact test. Factors that showed statistically significant results in the univariable analysis (p-value<0.05) were further explored using multivariable logistic regression analysis.

Results

The prevalence of uncontrolled type 2 diabetes was 29.1%. Multivariable analysis revealed that DRD (OR 14.20, 95% CI 3.76-53.64, p<0.001), age under 65 years old (OR 6.40, 95% CI 2.07-19.77, p=0.001), and obesity (BMI ≥ 25) (OR 2.96, 95% CI 1.05-8.39, p=0.041) were significantly associated with uncontrolled type 2 diabetes. Three dimensions of DRD were associated with uncontrolled type 2 diabetes, including emotional distress (OR 4.23, 95% CI 1.51-11.85, p=0.006), regimen-related distress (OR 6.00, 95% CI 1.88-19.18, p=0.003), and interpersonal distress (OR 5.25, 95% CI 1.39-20.02, p=0.015).

Commented [DZABZ1]: I would recommend to use a general term i.e., 'factors' here

Commented [DZABZ2]: Please consider rephrasing. It would be clearer if DRD is shown to be linked to poor glycemic control.

Commented [DZABZ3]: Prevalence was not in the title.

Commented [DZABZ4]: Personally, I do not think that this is a valid design as prospective usually involves some degree of follow-up while cross-sectional study does not.

I shall leave this up to the discussion among your team to decide.

Commented [DZABZ5]: Consider rephrasing the sentence as t-test does not measure association between factors.

Conclusion and recommendation

Age, obesity, and DRD are associated with uncontrolled type 2 diabetes. Highlights the need for a holistic approach that considers both physical and psycho-social factors to prevent diabetic complications. Further studies with broader populations using a cohort-based design are recommended.

Keywords:

uncontrolled diabetes, diabetes related distress, factor, behavior, psychosocial, DDS

Introduction

Type 2 diabetes mellitus (DM) is a major global health concern with a high prevalence of 462 million cases in 2017 [1], and is expected to rise to 643 million by 2030 [2]. Uncontrolled diabetes can cause many serious complications including retinopathy, nephropathy, neuropathy, and cardiovascular diseases [3], which impact the quality of life, increasing mortality and raising healthcare expenditures [4]. Effective glycemic control is importance to prevent diabetic complications. To ensure this, guidelines have been continuously updated to focus on maintaining optimal glycemic levels. However, poor glycemic control remains widespread, i.e., the prevalence of uncontrolled diabetes was 47.3% in Brazil [5], 50.2% in Japan [6], and 76.9% in Saudi Arabia [7]. Thailand ranks fifth in the number of DM cases among Western Pacific countries [2], with 64.4% of DM cases being uncontrolled [8].

Achieving a glycemic goal is difficult due to numerous factors, which can be divided into 3 aspects: physical, behavioral, and psycho-social aspect. In the physical aspect, there are many factors associated with uncontrolled diabetes including age [8, 9, 10], sex [8], obesity [11, 12], duration of diabetes [7, 8], and diabetes treatment modalities [5, 10]. In the behavioral aspect, factors associated with uncontrolled diabetes including self-care behaviors [11], energy intake [13], physical activities [5, 14, 15], sleeping quality [16], and smoking [7]. In the psycho-social aspect, depression is a common psychological issue associated with uncontrolled diabetes [17]. However, evaluation for depression does not cover the social aspect, which is also important for diabetes treatment outcomes. So, diabetes-related distress (DRD) [18, 19, 20] is a new trending concern.

Diabetes-related distress (DRD) refers to a broader affective experience than Depression. It covers both psychological and social aspects of….. DRD implies worries, concerns, and fears related to the burden of living with diabetes, such as feeling overwhelmed by diabetes management tasks or experiencing negative emotions related to the condition [21]. DRD is more common in diabetic patients than depression [22], with a prevalence of 29.4% in Vietnam [23], 37.0% in Saudi Arabia [18], and 39% in Canada [24]. Furthermore, DRD is associated with poor self-management [18, 20] and poor glycemic outcomes [18, 19, 20]. Despite the importance of DRD in diabetes management, it is often overlooked in clinical settings. Therefore, attending to psycho-social concerns may be the key to achieve the glycemic goal. DRD screening may be an important step in dealing with psycho-social problems in diabetic patients.

Literature review shows well-studied physical and behavioral factors affecting glycemic control in patients with type 2 diabetes. However, there is a gap in knowledge regarding the psycho-social factors in Thailand. Especially, the impact of DRD, which includes both psychological and social aspect, has not been well studied. Therefore, this study

Commented [DZABZ6]: Consider adding a statistic related to Northern Thailand, if any. Personally, I think it would be interesting to see if the identified factors are only true for diabetics residing in certain part of Thailand.

Commented [DZABZ7]: I strongly believe that age and sex are demographic factors rather than physical factors. Please consider rephrasing or re-classifying the factors.

Commented [DZABZ8]: These factors sound more like clinical factors rather. Please consider re-classifying.

Commented [DZABZ9]: Consider using a conjunction that is more appropriate for scientific writing.

Deleted: ,

Deleted: i

Commented [DZABZ10]: I do not quite get what this sentence means. Please consider rephrasing.

Commented [DZABZ11]: Consider adding this statement in the abstract.

aims to investigate the prevalence and associated factors of uncontrolled type 2 diabetes, including DRD, in Maharaj Nakorn Chiang Mai Hospital, Chiang Mai, Thailand.

Materials and methods

Study design, setting, and period

A cross-sectional study was conducted from 1st December 2021 to 18th March 2022 at the Outpatient Clinic of the Family Medicine Department, Maharaj Nakorn Chiang Mai Hospital, Chiang Mai, Thailand. Maharaj Nakorn Chiang Mai Hospital, affiliated with the Faculty of Medicine, Chiang Mai University, functions as both a training center and a tertiary medical care provider for patients in Chiang Mai and 16 other Northern Thai provinces. The hospital's Family Medicine Department's Outpatient Clinic serves as a primary care facility for non-urgent medical conditions. The clinic registers and treats about 9,000 patients each year, with around 1,9000 cases being type 2 diabetes patients.

Population

The study population consisted of type 2 diabetic patient registered in the Outpatient Clinic of the Family Medicine Department, Maharaj Nakorn Chiang Mai Hospital.

Inclusion criteria were: 1) type 2 diabetic patients who visited during the study period 2) age at least 18 years old 3) be able to answer the questionnaire and agree to participate voluntarily. Exclusion criteria were: 1) being new diagnosis type 2 diabetes, 2)unwillingness to participate the study.

Sampling and sample size estimation

Total of 127 patients were recruited by consecutive sampling. Sample size was calculated by using Estimating and infinite population proportion formula [25] based on proportion of 50.2% of uncontrolled type 2 diabetes(5), error(d)= 0.10, alpha = 0.05, Z(0.975) = 1.96. Thus, the calculated sample size was 96 patients. Moreover, there were 20% added to prevent data collection errors.

Instrument and data collection

Physical aspect data was collected through a questionnaire interviewed by one research data collection assistant, including age, gender, occupation, income, education, marital status, health insurance, comorbidities, and duration of being type 2 diabetes. Medical characteristic data including weight, height, waist circumference, blood pressure, fasting blood sugar (FBS), glycated hemoglobin (HbA1C) and diabetes treatment modalities were obtained from medical record. Body Mass Index (BMI) is calculated by dividing a person's weight in kilograms by their height in meters squared. In this study, a BMI of ≥ 25 kg/m^2 was considered as an indicator of obesity [26].

For behavioral factors, smoking and alcohol consumption was collected via questionnaire. Self-care behavior was assessed using a questionnaire developed by Siangdung S. (Cronbach's ɑ=0.70) [27]. This questionnaire consists of 20 questions covering 5 behaviors including eating, medical adherence, exercise, dealing with stress and continuing of treatment. Each question is rating scale:1 (not performing), 2 (perform occasionally), and 3 (perform regularly). The mean score of behavior was categorized into 3 groups of self-care: low (1.00-1.66), medium (1.67-2.33) and high (2.34-3.00). Physical activity was collected using Thai version Global Physical Activity Questionnaires (GPAQ) (Cronbach's ɑ=0.82) [28, 29]. Which recorded physical activity data and calculated to metabolic equivalent (MET). WHO recommended that adequate physical activity required at least 150 minutes of moderate or 75 minutes of vigorous intensity physical activity per week or at least 600 MET [29]. Dietary assessment was collected using 24-hour diet recall. The research assistant instructed the patient to provide information about the type, name, and amount of food and

Commented [DZABZ12]: I would suggest using these terms in the manuscript title.

Commented [DZABZ13]: I have a few major concerns here. If one of the objectives is to identify the prevalence of uncontrolled T2DM, I believe there should be a sub-group analysis on those with uncontrolled DM.

If we remove prevalence from the main objective, technically, after screening, only patients with documented uncontrolled DM should be included in this study. Then only, factors associated with uncontrolled DM can be further assessed.

Commented [DZABZ14]: It would be better to specify, if a patient visited the clinic more than once during the 1-year data collection period, which data was collected for analyses?

Commented [DZABZ15]: In my opinion, the duration has to be more specific as this it could influence many variables in the study e.g., DRD, glycemic control etc.

Commented [DZABZ16]: This is an automatic exclusion. Please consider to remove.

Commented [DZABZ17]: Please be more specific.

Commented [DZABZ18]: As there were quite a number of scores used, please clarify whether the research assistant has undergone any training/briefing to administer the questionnaire. I am particularly concerned about the depression rating scale.

It would also be better to clearly indicate which scoring tool is self-administered vs interviewer-assisted.

Commented [DZABZ19]: Please specify. Monthly or yearly? Household or patient's own income?

Commented [DZABZ20]: As commented before, the factors are mixed of demographic and clinical factors. Please consider re-classifying the factors.

Deleted: riage

drink consumed in the previous 24 hours. Total calorie and carbohydrate intake were analyzed by a dietitian, while other data was kept blind to prevent bias. Sleep quality in the past 1 month was evaluated using Thai version Pittsburgh Sleep Quality Index (PSQI) (Cronbach's ɑ=0.84) [30] This questionnaire contained 19 questions which are interpreted to total score 0-21, score of ≥ 5 indicating poor sleep quality.

To evaluate psycho-social aspect, depression screening was assessed using 9-Questions Depression Rating Scale (9Q) [31]. This screening tool consists of 9 questions about depressive symptoms experienced within the last 2 weeks, scoring ≥ 7 means 9Q positive result for depression. Diabetes-related distress was assessed using Thai version Diabetes Distress Scale questionnaire (DDS-17) (Cronbach's ɑ=0.95) [22, 32]. This questionnaire consists of 17 questions in four different dimensions (emotional distress, regimen-related distress, physician-related distress, and diabetes-related interpersonal distress). Each question is rating scale from 1 (no problems) to 6 (serious problems). Each dimension was interpreted by its mean score into 2 groups: no distress (< 2), with distress (moderate distress (2-2.9) and high distress (≥ 3)). The author had access to information that could identify individual participants during data collection.

Outcome of interest

Glycemic control was determined according to The American Diabetes Association (ADA) Guideline 2021 [33]. The target HbA1C was determined based on age and comorbidities, including: 1) HbA1C < 7.0% (age ≤ 65 years old) 2) HbA1C < 7.5% (age > 65 years old, independent, without cognitive impairment) 3) HbA1C < 8.0% (age > 65 years old with ≥ 3 comorbidities (e.g., hypertension, arthritis, cancer, heart failure, falls, CKD stage ≥ 3, myocardial infarction, stroke), frailty, dementia, dependent).

Statistical analysis

Data was analyzed by using Stata version 16.0 (StataCorp, College Station, Texas, USA). Descriptive statistics are expressed as frequencies and percentage for categorical data, mean ± SD or median (IQR) for numerical data according to the underlying distribution. Independent t-test and Fisher's exact test were used to test for the univariable association between potentially related clinical characters and uncontrolled type 2 diabetes. Factors with statistically significant results from univariable analysis (p-value < 0.05) were furthered explored using multivariable logistic regression analysis.

Ethical considerations

Ethical approval was obtained from the Research Ethics Committee, Faculty of Medicine, Chiang Mai University, Thailand (FAM-2564-08500). Written informed consent was obtained from the study participants after informing the purpose of the study.

Results

Total of 160 diabetes patients who visit Outpatient Clinic of the Family Medicine Department, Maharaj Nakorn Chiang Mai Hospital during the study performed. There were 33 patients who were excluded, 4 patients were newly diagnosis of type 2 diabetes, and 29 patients were not voluntary to participate the study. Therefore, there were 127 patients who voluntareed to participate in this study and met the inclusion criteria,

The mean age was 66.2 ± 7.3 years, with female predominance (52%). Most of them were married and live with their family (73.2%). The mean HbA1C was 6.9 ± 0.8% and the prevalence of uncontrolled type 2 diabetes was 29.1%. Table 1 shows the comparison of physical factors between uncontrolled and controlled type 2 diabetes. The uncontrolled group had higher prevalence of age under 65 years old (p-value=0.014) and obesity (p-

Commented [DZABZ21]: As per commented before

Commented [DZABZ22]: There were statistically non-significant factors evaluated using regression analyses as well e.g., age

Deleted: ry

Deleted: .

value=0.012), using sulfonylurea (p-value=0.029) and using two or more types of diabetes medication (p-value=0.012). (**Table 1)**

Table 1 Comparison of physical characteristics between uncontrolled and controlled type 2 diabetes (n=127)

Factor	DM treatment outcome				p-value
	Uncontrolled (n=37)		Controlled (n=90)		
	n	%	n	%	
Personal characteristics					
Age					
Age < 65 years old	19	51.4	25	27.8	
Age ≥ 65 yearsold (elderly)	18	48.6	65	72.2	0.014
Sex					
Male	16	43.2	45	50.0	
Female	21	56.8	45	50.0	0.560
Occupation					
Have a job	16	43.2	32	35.6	0.428
No job/Pensioner	21	56.8	58	64.4	0.428
Income (median (IQR))	20000	(10000,32000)	16384	(10000,30000)	0.280
Education level					
Lower than bachelor's degree	20	54.1	50	55.6	1.000
Bachelor's degree or higher	17	46.0	40	44.4	1.000
Married, live with family	26	70.3	67	74.4	0.663
Health Insurance					
Government	36	97.3	85	94.4	
Non-government	1	2.7	5	5.6	0.671
Medical characteristics					
Comorbidities					
> 1 other underlying diseases	30	81.1	81	90.0	0.237
Body weight (kg) (mean ± SD)	65.6	± 11.5	63.5	± 12.0	0.378
Height (cm) (mean ± SD)	157.8	± 8.3	159.4	± 8.5	0.329
Obesity (BMI ≥ 25 kg/m2)	24	64.9	36	40.0	0.012
Waist circumference (cm) (mean ± SD)	88.4	± 9.5	87.4	± 10.0	0.621
Systolic blood pressure(mmHg) (mean ± SD)	140.5	± 10.7	136.5	± 13.1	0.098
Diastolic blood pressure(mmHg)(mean± SD)	79.9	± 8.8	78.4	± 9.4	0.422
Diabetes characteristics					
Duration of DM (years) (median (IQR))	9	(4,10)	8	(3,11)	0.845
Diabetes treatment					
Lifestyle modification	4	10.8	11	12.2	1.000
Biguanide	32	86.5	76	84.4	1.000
Sulfonylureas	15	40.5	19	21.1	0.029
Thiazolidinedione	2	5.4	3	3.3	0.628
DPP-4 inhibitor	15	40.5	33	36.7	0.692
SGLT-2 inhibitor	2	5.4	2	2.2	0.579
Insulin	0	0	1	1.1	1.000
Using ≥ 2 types of DM medication	24	64.9	36	40.0	0.012
FBS (mg/dL) (mean ± SD)	155.8	± 35.0	122.9	± 16.2	< 0.001
HbA1C (%) (mean ± SD)	7.8	±0.6	6.5	±0.5	<0.001

Concerning behavioral and psycho-social factors, most patients had high level of self-care behavior (84.3%), while 22.1% of them had inadequate physical activity and 37.7% experienced poor sleep quality. The prevalence of depression and diabetic-related distress among patients in this study were 6.3% and 19.7%, respectively. Table 2 presents the comparison of behavioral and psycho-social factors between the uncontrolled and controlled type 2 diabetes. In terms of behavioral factors, there were no significant differences in self-care behavior, physical activity, dietary intake, smoking, and alcohol consumption between

Commented [DZABZ23]: Please indicate the type of statistics use and write it at the bottom of the table.

Commented [DZABZ24]: Patients in the controlled group has lower mean body weight and higher height. However, there were of them who are obese. It does not look right. Please double check the numbers.

the two groups. Interestingly, psycho-social factors were particularly remarkable. The uncontrolled group had higher prevalence of being diabetic patient with distress (p-value<0.001), depression (p-value=0.046), and poor sleep quality (p-value=0.008). (**Table 2**)

Table 2 Comparison of behaviors and psycho-social factors between uncontrolled and controlled type 2 diabetes (n=127)

Factor	DM treatment outcome				p-value
	Uncontrolled (n=37)		Controlled (n=90)		
	n	%	n	%	
**Behavioral factors**					
Current smoking	4	10.8	2	2.2	0.059
Current alcohol drinking	13	35.1	20	22.2	0.181
Self-care behavior (Total Health Behavior score) (mean ± SD)	2.5	± 0.2	2.6	± 0.2	0.221
High (score 2.34-3.00)	31	83.8	76	84.4	
Medium (score 1.67-2.33)	6	16.2	14	15.6	
Low (score 1.00-1.66)	0	0	0	0	1.000
Physical activity (MET score) (median (IQR))	880	(560,2860)	1120	(720,2120)	0.538
Inadequate physical activity	11	29.7	17	18.9	0.238
24 hr. Dietary Recall					
Carbohydrate (kcal/day) (mean ± SD)	829.9	±347.4	851.9	±332.7	0.738
Total calory (kcal/day) (mean ± SD)	1598.6	±463.9	1657.3	±510.0	0.546
Sleep quality by PSQI score					
Poor sleep quality (PSQI >5)	21	56.8	27	30.0	0.008
**Psycho-social factors**					
Screening Depression by 9Q					
Positive (9Q ≥ 7)	5	13.5	3	3.3	0.046
DRD by DDS-17 score					
No distress (score <2)	18	48.7	84	93.3	
With distress (score ≥ 2)	19	51.4	6	6.7	< 0.001

Table 3 presents Multivariable analysis between uncontrolled type 2 diabetes and potential related factors. Factors significantly associated with uncontrolled type 2 diabetes were age under 65 years old (p-value=0.001), obesity (p-value=0.041), and being diabetic patient with distress (p-value<0.001). (**Table 3**)

Table 3 Multivariable analysis of association between uncontrolled type 2 diabetes and potential related factors (n=127)

Factor	Odd Ratio	95% CI	p-value
Age			
Age ≥ 65 years old (elderly)	Reference		
Age < 65 years old	6.40	2.07-19.77	0.001
Sex			
Male	Reference		
Female	1.66	0.58-4.77	0.343
BMI			
Not obesity (BMI < 25)	Reference		
Obesity (BMI ≥ 25)	2.96	1.05-8.39	0.041
Using sulfonylureas			
Not using sulfonylureas	Reference		
Using sulfonylureas	1.91	0.52-7.09	0.331
DM medication			

Commented [DZABZ25]: Please indicate the type of statistics use and write it at the bottom of the table.

Commented [DZABZ26]: Was the amount of alcohol consumed measured?

Lifestyle modification or using 1 type of DM medication	Reference		
Using two or more types of diabetes medication	1.89	0.55-6.47	0.313
Diabetes-related distress (DRD)			
No distress (score < 2)	Reference		
With distress (score ≥ 2)	14.20	3.76-53.64	<0.001
Depression screening (9Q)			
No depression (9Q < 7)	Reference		
Depression (9Q ≥ 7)	2.88	0.31-26.33	0.349
Sleep quality (PSQI)			
Normal sleep quality (PSQI ≤ 5)	Reference		
Poor sleep quality (PSQI > 5)	2.01	0.66-6.14	0.219

Table 4 presents the comparison of Diabetes Distress Scale (DDS-17) dimensions between uncontrolled and controlled type 2 diabetes. The prevalence of emotional distress was 26.0%, regimen-related distress was 22.1%, physician-related distress was 3.2%, and diabetes-related interpersonal distress was 12.6%. Moreover, the uncontrolled group had higher prevalence of emotional distress (p-value<0.001), regimen-related distress (p-value<0.001) and diabetes-related interpersonal distress (p-value=0.001). (**Table 4**)

Table 4 Comparison of DDS-17 dimensions between uncontrolled and controlled type 2 diabetes (n=127)

Factor	DM treatment outcome				p-value
	Uncontrolled (n=37)		Controlled (n=90)		
	n	%	n	%	
Emotional distress					
No distress (score <2)	18	48.7	76	84.4	
With distress (score ≥ 2)	19	51.3	14	15.6	<0.001
Regimen-related distress					
No distress (score <2)	19	51.3	80	88.9	
With distress (score ≥ 2)	18	48.7	10	11.1	<0.001
Physician-related distress					
No distress (score <2)	35	94.6	88	97.8	
With distress (score ≥ 2)	2	5.4	2	2.2	0.579
Diabetes-related interpersonal distress					
No distress (score <2)	26	70.3	85	94.4	
With distress (score ≥ 2)	11	29.7	5	5.6	0.001

Table 5 presented multivariable analysis of association between uncontrolled type 2 diabetes and DDS-17 dimensions. Emotional distress (p-value=0.006), regimen-related distress (p-value=0.003), and diabetes-related interpersonal distress (p-value=0.015) were associated with uncontrolled type 2 diabetes after adjusted for age, sex, obesity, using sulfonylureas, using two or more types of diabetes medication, depression, and poor sleep quality. (**Table 5**)

Table 5 Multivariable analysis of association between uncontrolled type 2 diabetes and DDS-17 dimensions (n=127)

Factor	Odd Ratio	95% CI	p-value
Emotional distress			
No distress (score <2)	Reference		
With distress (score ≥ 2)	4.23	1.51-11.85	0.006
Regimen-related distress			
No distress (score <2)	Reference		

With distress (score ≥ 2)	6.00	1.88-19.18	0.003
Physician-related distress			
No distress (score <2)	Reference		
With distress (score ≥ 2)	1.22	0.11-13.23	0.869
Diabetes-related interpersonal distress			
No distress (score <2)	Reference		
With distress (score ≥ 2)	5.25	1.38-20.02	0.015

Adjust for age, sex, obesity, using sulfonylureas, using two or more types of diabetes medication, depression, and poor sleep quality.

Discussion

The purpose of this study was to investigate the prevalence and identify factors associated with uncontrolled type 2 diabetes in Maharaj Nakorn Chiang Mai Hospital, Chiang Mai, Thailand. In our study, the prevalence of uncontrolled type 2 diabetes was 29.1%. Univariable analysis revealed that factors associated with uncontrolled type 2 diabetes were being under 65 years old, obesity, using sulfonylureas, using two or more types of diabetes medication, depression, being diabetic patient with distress, and poor sleep quality. According to multivariable analysis, being under 65 years old, obesity, and being diabetic patient with distress were associated with uncontrolled type 2 diabetes. These findings emphasized the importance of considering both physical and psychosocial factors when assessing and managing glycemic control in patients with uncontrolled type 2 diabetes.

Commented [DZABZ27]: Based on the data and statistical analyses used, I do not think that the main findings are accurate. This is because only 37 patients in this study had uncontrolled DM whereas the other 90 patients had controlled DM.

The results have to be rephrased to 'factors associated with control of DM', instead.

Please kindly discuss further with your team.

The prevalence of uncontrolled type 2 diabetes in our study was found to be lower than in the previous studies, such as 47.3% in Brazil [5], 50.2% in Japan [6], and 84.3% in Uganda [9]. The difference in prevalence might be due to variations in the HbA1C goal, some countries use an HbA1C goal of less than 7 mg/dl [6, 9], and some countries use an HbA1C goal according to ADA guideline 2021 [5], which allows greater flexibility for the older patients. Additionally, sociodemographic variables and the healthcare setting at Family Medicine OPD as a setting in a university hospital that had educational activities and many facilities such as multidisciplinary care and holistic care might have contributed to the lower prevalence of uncontrolled type 2 diabetes [5].

Multivariable analysis in this study found 3 factors significantly associated with uncontrolled type 2 diabetes. The first was being under 65 years old, consistent with findings from previous studies [8, 9, 10, 34]. This could be due to younger to middle-aged patients might be more occupied with work and more likely to miss doctor's appointments and disregard self-care behaviors, leading to poor glycemic control [8, 9, 34]. Conversely, elderly patients might be more motivated to take care of their diabetic conditions [8]. Additionally, this might be the result of the flexible HbA1C target range for the elderly in the ADA guideline 2021 [33]. The second factor, obesity was also found to be associated with uncontrolled type 2 diabetes, which was consistent with previous studies [11, 12] that patients with greater BMI (obesity), who were expected to have low physical activity levels, were more likely to have poor glycemic control than those with normal BMI [11]. Furthermore, evidence supported the hypothesis that obesity was associated with insulin resistance and beta-cell dysfunction, which resulted in hyperglycemia [35]. Therefore, addressing obesity through weight reduction interventions could improve glycemic control and general health status in patients with uncontrolled type 2 DM [36].

The last factor was diabetes-related distress (DRD). The prevalence of diabetic patients with distress in this study was 19.7%, which was close to previous studies, including 22.6% in Greece [37], 25% in Saudi Arabia [38], and 29.4% in Vietnam [23]. However, another study conducted in Canada showed a higher prevalence of DRD, 39% [24]. This result showed that the prevalence of DRD varied among different countries and service

settings. Multivariable analysis in this study revealed a significant association between being diabetic patients with distress and uncontrolled type 2 diabetes. This finding was consistent with previous studies conducted in Japan [19], Saudi Arabia [18], and the United States [39]. Previous studies found that DRD, rather than depression, was associated with higher HbA1C levels [19, 20]. DRD was also associated with a low level of autonomy support [39], poor treatment adherence [18], and poor glycemic control [18, 39]. Moreover, evidence supported that DRD could lead to stress hormone dysregulation and subsequent hyperglycemia [37]. Regarding the subgroup analysis of DRD, it was found that emotional distress and regimen-related distress were common among type 2 diabetic patients in this study. The prevalence of emotional distress was 26.0%, regimen-related distress was 22.1%, physician-related distress was 3.2%, and diabetes-related interpersonal distress was 12.6%. The results of the multivariable analysis found significant association between emotional distress, regimen-related distress, and diabetes-related interpersonal distress with uncontrolled type 2 diabetes, which was consistent with previous studies [24, 37, 40]. Studies found association between emotional distress and poor glycemic control, which could be attributed to the psychological stress of managing their condition [24, 37, 40]. Furthermore, diabetes-related interpersonal distress was also found to be associated with poor glycemic control [37], this could be a result of the difficulties in maintaining social relationships and coping with diabetic condition's impact on daily life. Additionally, regimen-related distress was found to be associated with poor medication adherence [41] and higher HbA1C levels [24, 37], which could be related to the difficulties in adhering to their medication and self-care management. Besides, evidence supported that a lower level of DRD score was positively associated with better glycemic control [23]. Therefore, the DDS-17 could be used to assess patients' specific concerns and assist in personalized diabetes management plans. Additionally, studies revealed that interventions such as peer support and brief group cognitive behavioral therapy can effectively reduce DRD and improve glycemic control in uncontrolled type 2 diabetic patients with DRD [42, 43]. Consequently, appropriate DRD detection and management through screening and implementation of interventions, such as cognitive-behavioral therapy or supervision, could enhance self-care and improve treatment outcomes for diabetic patients.

In addition to the factors previously discussed, this study found other potential factors associated with uncontrolled type 2 diabetes through univariable analysis, including using two or more types of diabetes medication [10], depression [17], and poor sleep quality [16]. All these factors were supported by previous studies [10, 16, 17]. Depression and sleep quality were found to be potentially associated with uncontrolled type 2 diabetes. However, these factors were only found to be significant in the univariable analysis, possibly due to the small sample size.

The main strength of the study is a comprehensive evaluation of potential factors associated with uncontrolled type 2 diabetes in multiple dimensions, including physical, behavioral, and psychosocial factors. This approach provides a more holistic view of the factors associated with uncontrolled type 2 diabetes. Another strength of the study is the use of standardized tools for data collection and analysis, which enhanced the accuracy and reliability of the results. The expertise of a dietitian also improved the quality of the results particularly in dietary assessment. Moreover, the use of a single assistant to collect the data minimized inconsistency in data collection and potential bias. However, there are some limitations to this study. Firstly, the cross-sectional design of this study did not allow for confirmation of a causal relationship between potential factors associated with uncontrolled type 2 diabetes, including DRD and glycemic control. Further research should be cohort-based to confirm causality. Moreover, the study population was limited to patients at the OPD clinic of Family Medicine, Maharaj Nakorn Chiang Mai Hospital, which might limit the

Commented [DZABZ28]: True, but it has to be mentioned about the training that the assistant has undergone prior to collecting the data.

Commented [DZABZ29]: I would think that reporting bias would also be a limitation because the data from the interviews may not be accurate. Moreover, 83/127 patients were elderly patients with majority of them having > 1 comorbidities.

generalizability of the findings. To increase the generalizability of the results, future studies should include a more diverse population.

Conclusion

Despite a relatively low prevalence of uncontrolled type 2 diabetes in this study, it remains a problem that requires attention. This study identified several factors associated with uncontrolled type 2 diabetes, including being under 65 years old, obesity, and diabetes-related distress. Additionally, three dimensions of diabetes-related distress, including emotional distress, regimen-related distress and diabetes-related interpersonal distress were found to be associated with uncontrolled type 2 diabetes. This study emphasizes the need for a more holistic approach to diabetes management. To effectively achieve glycemic control and prevent diabetes-related complications, clinicians must consider not only the physical aspect but also psycho-social factors in their approach to patient care.

Acknowledgments

The authors thank the Family Medicine Department, Maharaj Nakorn Chiang Mai Hospital for their support, the OPD family medicine staff for their help in patient recruitment, and all participants for providing informative data and giving permission for its publication.

References

1. Khan MAB, Hashim MJ, King JK, Govender RD, Mustafa H, Al Kaabi J. Epidemiology of Type 2 Diabetes - Global Burden of Disease and Forecasted Trends. J Epidemiol Glob Health. 2020;10(1):107-11.
2. International Diabetes Federation. IDF Diabetes Atlas 10th ed. Brussels, Belgium: International Diabetes Federation; 2021. p. 5-6.
3. Banday MZ, Sameer AS, Nissar S. Pathophysiology of diabetes: An overview. Avicenna J Med. 2020;10(4):174-88.
4. Reutrakul S, Deerochanawong C. Diabetes in Thailand: Status and Policy. Current Diabetes Reports. 2016;16(3):28.
5. Espinosa MM, Almeida V, Nascimento VFD. Poor glycemic control and associated factors in diabetic people attending a reference outpatient clinic in Mato Grosso, Brazil. Invest Educ Enferm. 2021;39(3).
6. Gouda M, Matsukawa M, Iijima H. Associations between eating habits and glycemic control and obesity in Japanese workers with type 2 Diabetes Mellitus. Diabetes Metab Syndr Obes. 2018;11:647-58.
7. Riaz F, Al Shaikh A, Anjum Q, Mudawi Alqahtani Y, Shahid S. Factors related to the uncontrolled fasting blood sugar among type 2 diabetic patients attending primary health care center, Abha city, Saudi Arabia. Int J Clin Pract. 2021;75(7):e14168.
8. Sakboonyarat B, Pima W, Chokbumrungsuk C, Pimpak T, Khunsri S, Ukritchon S, et al. National trends in the prevalence of glycemic control among patients with type 2 diabetes receiving continuous care in Thailand from 2011 to 2018. Scientific Reports. 2021;11(1):14260.
9. Patrick NB, Yadesa TM, Muhindo R, Lutoti S. Poor Glycemic Control and the Contributing Factors Among Type 2 Diabetes Mellitus Patients Attending Outpatient Diabetes Clinic at Mbarara Regional Referral Hospital, Uganda. Diabetes Metab Syndr Obes. 2021;14:3123-30.
10. Marczynski M, Cortellazzi K, Barberato-Filho S, Motta R, Vieira A, Quilici M, et al. Unsatisfactory glycemic control in type 2 Diabetes mellitus patients: predictive factors and negative clinical outcomes with the use of antidiabetic drugs. Brazilian Journal of Pharmaceutical Sciences. 2016;52:801-12.
11. Almomani MH, Al-Tawalbeh S. Glycemic Control and Its Relationship with Diabetes Self-Care Behaviors Among Patients with Type 2 Diabetes in Northern Jordan: A Cross-Sectional Study. Patient Prefer Adherence. 2022;16:449-65.

12. Boye KS, Lage MJ, Thieu V, Shinde S, Dhamija S, Bae JP. Obesity and glycemic control among people with type 2 diabetes in the United States: A retrospective cohort study using insurance claims data. J Diabetes Complications. 2021;35(9):107975.

13. Kang HM, Kim DJ. Total energy intake may be more associated with glycemic control compared to each proportion of macronutrients in the korean diabetic population. Diabetes Metab J. 2012;36(4):300-6.

14. Park JH, PhD, Lee YE, PhD. Effects of exercise on glycemic control in type 2 diabetes mellitus in Koreans: the fifth Korea National Health and Nutrition Examination Survey (KNHANES V). J Phys Ther Sci. 2015;27(11):3559-64.

15. Ahn J, Yang Y. Factors Associated with Poor Glycemic Control Amongst Rural Residents with Diabetes in Korea. Healthcare (Basel). 2021;9(4).

16. Tang Y, Meng L, Li D, Yang M, Zhu Y, Li C, et al. Interaction of sleep quality and sleep duration on glycemic control in patients with type 2 diabetes mellitus. Chin Med J (Engl). 2014;127(20):3543-7.

17. Papelbaum M, Moreira RO, Coutinho W, Kupfer R, Zagury L, Freitas S, et al. Depression, glycemic control and type 2 diabetes. Diabetol Metab Syndr. 2011;3(1):26.

18. Fayed A, AlRadini F, Alzuhairi RM, Aljuhani AE, Alrashid HR, Alwazae MM, et al. Relation between diabetes related distress and glycemic control: The mediating effect of adherence to treatment. Prim Care Diabetes. 2022;16(2):293-300.

19. Tsujii S, Hayashino Y, Ishii H. Diabetes distress, but not depressive symptoms, is associated with glycaemic control among Japanese patients with type 2 diabetes: Diabetes Distress and Care Registry at Tenri (DDCRT 1). Diabet Med. 2012;29(11):1451-5.

20. Fisher L, Glasgow RE, Strycker LA. The relationship between diabetes distress and clinical depression with glycemic control among patients with type 2 diabetes. Diabetes Care. 2010;33(5):1034-6.

21. Fisher L, Gonzalez JS, Polonsky WH. The confusing tale of depression and distress in patients with diabetes: a call for greater clarity and precision. Diabet Med. 2014;31(7):764-72.

22. Thanakwang K, Thinganjana W, Konggumnerd R. Psychometric properties of the Thai version of the Diabetes Distress Scale in diabetic seniors. Clin Interv Aging. 2014;9:1353-61.

23. Huynh G, Tran TT, Do THT, Truong TTD, Ong PT, Nguyen TNH, et al. Diabetes-Related Distress Among People with Type 2 Diabetes in Ho Chi Minh City, Vietnam: Prevalence and Associated Factors. Diabetes Metab Syndr Obes. 2021;14:683-90.

24. Wong EM, Afshar R, Qian H, Zhang M, Elliott TG, Tang TS. Diabetes Distress, Depression and Glycemic Control in a Canadian-Based Specialty Care Setting. Can J Diabetes. 2017;41(4):362-5.

25. Daniel WW. Biostatistics: A foundation of analysis in the health sciences. John Wiley&Sons. 1995:180.

26. Garvey WT, Mechanick JI, Brett EM, Garber AJ, Hurley DL, Jastreboff AM, et al. AMERICAN ASSOCIATION OF CLINICAL ENDOCRINOLOGISTS AND AMERICAN COLLEGE OF ENDOCRINOLOGY COMPREHENSIVE CLINICAL PRACTICE GUIDELINES FOR MEDICAL CARE OF PATIENTS WITH OBESITY. Endocr Pract. 2016;22 Suppl 3:1-203.

27. Siangdung S. Self-Care Behaviors of Patients with Uncontrolled DM. The Southern College Network Journal of Nursing and Public Health. 2017;4(1):191-204.

28. Jaturapatporn D, Hathirat S, Manataweewat B, Dellow AC, Leelaharattanarak S, Sirimothya S, et al. Reliability and validity of a Thai version of the General Practice Assessment Questionnaire (GPAQ). J Med Assoc Thai. 2006;89(9):1491-6.

29. World Health Organization. Global Physical Activity Questionnaire (GPAQ) Analysis Guide Switzerland: World Health Organization; [updated February 2019; cited 2022 Apirl]. Available from: https://www.who.int/docs/default-source/ncds/ncd-surveillance/gpaq-analysis-guide.pdf?sfvrsn=1e83d571_2.

30. Sitasuwan T, Bussaratid S, Ruttanaumpawan P, Chotinaiwattarakul W. Reliability and validity of the Thai version of the Pittsburgh Sleep Quality Index. J Med Assoc Thai. 2014;97 Suppl 3:S57-67.

31. Kongsuk T, Arunpongpaisal S, Janthong S, Prukkanone B, Sukhawaha S, Leejongpermpoon J. Criterion-Related Validity of the 9 Questions Depression Rating Scale revised for Thai Central Dialect. J psychiatr Assoc Thaolanf. 2018;63(4):321-44.

32. Polonsky WH, Fisher L, Earles J, Dudl RJ, Lees J, Mullan J, et al. Assessing psychosocial distress in diabetes: development of the diabetes distress scale. Diabetes Care. 2005;28(3):626-31.
33. American Diabetes Association. 12. Older Adults: Standards of Medical Care in Diabetes—2021. Diabetes Care. 2020;44(Supplement_1):S168-S79.
34. Yeemard F, Srichan P, Apidechkul T, Luerueang N, Tamornpark R, Utsaha S. Prevalence and predictors of suboptimal glycemic control among patients with type 2 diabetes mellitus in northern Thailand: A hospital-based cross-sectional control study. PLoS One. 2022;17(1):e0262714.
35. Al-Goblan AS, Al-Alfi MA, Khan MZ. Mechanism linking diabetes mellitus and obesity. Diabetes Metab Syndr Obes. 2014;7:587-91.
36. Leitner DR, Frühbeck G, Yumuk V, Schindler K, Micic D, Woodward E, et al. Obesity and Type 2 Diabetes: Two Diseases with a Need for Combined Treatment Strategies - EASO Can Lead the Way. Obes Facts. 2017;10(5):483-92.
37. Kintzoglanakis K, Gkousiou A, Vonta P, Sagmatopoulos A, Copanitsanou P. Depression, anxiety, and diabetes-related distress in type 2 diabetes in primary care in Greece: Different roles for glycemic control and self-care. SAGE Open Medicine. 2022;10:20503121221096605.
38. Aljuaid MO, Almutairi AM, Assiri MA, Almalki DM, Alswat K. Diabetes-Related Distress Assessment among Type 2 Diabetes Patients. J Diabetes Res. 2018;2018:7328128.
39. Lee AA, Piette JD, Heisler M, Rosland AM. Diabetes Distress and Glycemic Control: The Buffering Effect of Autonomy Support From Important Family Members and Friends. Diabetes Care. 2018;41(6):1157-63.
40. Ogbera A, Adeyemi-Doro A. Emotional distress is associated with poor self care in type 2 diabetes mellitus. J Diabetes. 2011;3(4):348-52.
41. Cummings DM, Lutes L, Littlewood K, DiNatale E, Hambidge B, Schulman K, et al. Regimen-Related Distress, Medication Adherence, and Glycemic Control in Rural African American Women With Type 2 Diabetes Mellitus. Ann Pharmacother. 2014;48(8):970-7.
42. Mizokami-Stout K, Choi H, Richardson CR, Piatt G, Heisler M. Diabetes Distress and Glycemic Control in Type 2 Diabetes: Mediator and Moderator Analysis of a Peer Support Intervention. JMIR Diabetes. 2021;6(1):e21400.
43. Tunsuchart K, Lerttrakarnnon P, Srithanaviboonchai K, Likhitsathian S, Skulphan S. Benefits of Brief Group Cognitive Behavioral Therapy in Reducing Diabetes-Related Distress and HbA1c in Uncontrolled Type 2 Diabetes Mellitus Patients in Thailand. Int J Environ Res Public Health. 2020;17(15).

Supporting Information

Click here to access/download
Supporting Information
Funding_DM-DDS_14-3-2023.docx
