# Supplementary material for: Prevalence and the association between clinical factors and Diabetes-Related Distress (DRD) with poor glycemic control in patients with type 2 diabetes: A Northern Thai cross-sectional study
Source: PLoS One. 2023 Nov 27;18(11):e0294810. doi: 10.1371/journal.pone.0294810 (PMC10681199; doi:10.1371/journal.pone.0294810)
Supplement: Attachment — Submitted filename: response to reviewer2.docx [file pone.0294810.s003.docx]

Reviewers’ comment to the manuscript [PONE-D-23-07649]

entitled “Physical, behavioral, and psychosocial factors associated with uncontrolled type 2 diabetes: a Northern Thai cross-sectional study.”

Dear Editor and reviewers,

We would like to thank you for the opportunity to revise our manuscript to be qualified for publication PLOS ONE. We have revised and modified some parts of our manuscript as addressed in response to reviewers’ comments. We hope that our responses and revisions would substantially improve the quality of our manuscript and would be qualified for publication in the journal. If there were any further questions or minor points to be addressed or elaborated, please let us know. We would be more than eager to make any further revision.

Title

The use of the word “Physical factor” in this manuscript may be misleading and not according to usual diabetes mellitus literature. Please consider using conventional terminology.

- **Response:** Thank you for your suggestion. We edit title to “Prevalence and associated factors of poor glycemic control type 2 diabetes: a Northern Thai cross-sectional study.” (In revised manuscript without track change, line 1-2)

Abstract

Aside from “lack of studies,” please justify the need to study DRD in Thai diabetic population using other reasons.

Please use the signpost method: A→B, B→C, C→D, in order to write a cohesive background. At present, the background consists of 3 disjointed/non-cohesive sentences.

Please re-consider the usage of the words “physical data” and use “sociodemographic, clinical, etc. data” instead. “Diabetic information” can be rephrased as “disease-related information” or simply “clinical data”.

Please re-write the conclusion with better English sentence structure.

- **Response:** Follow your recommendation, we re-write abstract.
  - We re-write the background as follow: “Glycemic control is important to prevent diabetic complication. However, evidence linking factors such as diabetic-related distress (DRD) to poor glycemic outcomes is lacking in Thailand. Therefore, this study aimed to investigate the prevalence and associated factors of poor glycemic controlled type 2 diabetes.” (In revised manuscript without track change, line 19-22)
  - We and change the word physical data to demographic data and clinical data as follow: “Data collection included demographic data, clinical data (duration of type 2 DM, diabetic treatment modalities, weight, height, blood pressure, FBS, and HbA1C)” (In revised manuscript without track change, line 26-28)
  - We re-write conclusion: “Age, obesity, and DRD are associated with poor glycemic control. A holistic approach that includes addressing DRD is crucial for improving glycemic outcomes in patients with type 2 diabetes. Further studies with broader populations using a cohort-based design are recommended.” (In revised manuscript without track change, line 45-48)

Introduction

Line 66 – Please improve sentence to “is of importance” or “is important”

- **Response**: Thank you for your suggestion. We edit to “is important” as follow: “Effective glycemic control is important to prevent diabetic complications.” (In revised manuscript without track change, line 58-59)

Line 73 – Please justify the use of “physical factors” to denote age, sex, BMI, duration of diabetes and treatment modalities. None of the cited references that were included in the reference list had used the term “physical factors.” Physical factors usually denote physical activity or physical environment. Please go through the literature and use conventional definitions such as demographics, socioeconomics, sociodemographic, clinical, lifestyle-related, disease-related etc..

- **Response**: Following your recommendation, we edit the word physical factors to demographic and clinical factors as follow
  - “For demographic aspect, factors associated with poor glycemic control include age [8, 10, 11] and sex [8]. For clinical aspect, there are many factors associated with poor glycemic control, including obesity [12, 13], duration of diabetes [7, 8], and diabetes treatment modalities [5, 11].” (In revised manuscript without track change, line 67-71)

Line 81 – Please consider removing the word “trending”. Two out of the 3 citations ranged from earlier publications i.e. year 2010 and year 2012, implicating that diabetes-related distress is not a new concern.

- **Response**: Following your suggestion, we edit to as follow: “Therefore, diabetes related distress (DRD) is one of the important concerns[19, 20, 21].” (In revised manuscript without track change, line 75-76)

Line 82 – The introduction is DRD heavy, with a whole dedicated paragraph to it. It is also one of the objectives and makes up one-third of the results, with its own logistic regression table. It deserves an emphasis whether in the title or in the objective.

- **Response**: we add DRD in our objective as follow: “Therefore, this study aims to investigate: 1) the prevalence and associated factors of poor glycemic control type 2 diabetes, and 2) the prevalence of diabetes-related distress (DRD) and the association of its subcomponents and poor glycemic control type 2 diabetes in Maharaj Nakorn Chiang Mai Hospital, Chiang Mai, Thailand.” (In revised manuscript without track change, line 91-94)

Line 97 – Please separate between the prevalence of uncontrolled type-2 diabetes mellitus and prevalence of risk factors for uncontrolled type-2 diabetes mellitus. At present, the objectives are not stated clearly enough.

- **Response**: we edit our objective as follow: “Therefore, this study aims to investigate: 1) the prevalence and associated factors of poor glycemic control type 2 diabetes, and 2) the prevalence of diabetes-related distress (DRD) and the association of its subcomponents and poor glycemic control type 2 diabetes in Maharaj Nakorn Chiang Mai Hospital, Chiang Mai, Thailand.” (In revised manuscript without track change, line 91-94)

Line 93 & 94 – Please support your sentence with several citations.

- **Response**: we add several citations as suggested. “While literature has extensively studied demographic, clinical, and behavioral factors affecting glycemic control in patients with type 2 diabetes [5, 7, 8, 10, 11, 12, 13, 14, 15, 16, 17], there is a knowledge gap concerning psycho-social factors in Thailand.” (In revised manuscript without track change, line 87-89)

Materials and methods

Line 113 – Please state whether all type 2 diabetic patients are recruited, regardless of whether they are receiving pharmacological management or not.

- **Response**: In our study, all type 2 diabetic patients were recruited as described in the methods: “The patients received treatment based on routine guidelines, which include both medication with lifestyle modification and lifestyle modification only.” (In revised manuscript without track change, line 111-113)

Line 115 – Please clarify the definition of newly diagnosed i.e., 1 month? 1 year? etc. Please omit automatic exclusions such as “unwillingness to answer…” because your inclusion criteria require “willingness to answer.”

- **Response**:
  - In our study, definition of “Newly diagnosed” was diagnosed of type 2 DM within 6 months as described in exclusion criteria.
    - “Exclusion criterion was recent diagnosis of type 2 diabetes within the past 6 months.” (In revised manuscript without track change, line 116-117)
    - We omit “unwillingness to answer of exclusion criteria” as suggested. (In revised manuscript without track change, line 116-117)

Line 119 – Please justify the choice of this sample size formula. Please clarify whether the amount of diabetic outpatients were considered infinite or finite population. Please confirm whether the final sample size was sufficient for running a regression analysis.

- **Response**:
  - Our primary objective was to investigate the prevalence of poor glycemic control type 2 diabetes and we used infinite formula. For the question concerning whether the final sample size was sufficient for running a regression analysis, we add in the limitation part as follow: “As we did not perform an official sample size estimation for conducting the multivariable regression analysis, some important factors might not reach statistical significance. The interpretation of our results should focus on the direction and effect size rather than solely on the statistical significance, and further research with a larger study size is encouraged to explore relevant factors and validate our findings more comprehensively.” (In revised manuscript without track change, line 398-403)

Line 120 – Please state (cite) where the reference value of “50.2%” was obtained from and whether it was specific to Thailand/Northern Thailand. If not, please justify the selection of that particular value.

- **Response**: We have added a citation of a new reference value of 54.8%, which represents the prevalence of poor glycemic control in type 2 DM in Northern Thailand from a previous study, in the sample size estimation section: “Sample size was calculated by using Estimating and infinite population proportion formula [25] based on proportion of 54.8% of poorly controlled type 2 diabetes [9]” (In revised manuscript without track change, line 120-122)

Line 124 – Please state in detail whether the complete data collection process was conducted in one session or multiple small sessions. Please state who conducted the data collection, where did it take place. Was each section an individual interview or a patient self-reported questionnaire?

- **Response**: we describe in data collection part
  - Data collection was carried out within a single session.
  - Demographic data (age, sex, occupation, personal monthly income, education, marital status, and health insurance) was obtained from patients via a self-administered paper questionnaire.
  - Clinical data (comorbidities, duration of type 2 diabetes, cognitive screening test), behavioral data (smoking, alcohol consumption, self-care behavior, physical activity, dietary assessment, and sleep quality), and psycho-social data (depression screening and DRD) were obtained via questionnaire and assessment tool, interviewed by the trained research assistant nurse.
  - Remaining clinical data (weight, height, waist circumference, blood pressure, FBS, HbA1C, and diabetes treatment modalities) were obtained from the electronic medical records by another trained research assistant.
  - The detail of data collection process was added in method section as follow: “Data collection was carried out within a single session at the Outpatient Clinic of the Family Medicine Department, Maharaj Nakorn Chiang Mai Hospital. On the data collection day, all type 2 diabetic patients who visited the clinic and met the inclusion criteria were voluntarily invited to participate by the outpatient clinic's nurse. Participants willing to join the study received an explanation of the objectives and data collection process from another nurse, a specially trained research data collection assistant. Written informed consent was obtained on paper by the same assistant. Demographic data (age, sex, occupation, personal monthly income, education, marital status, and health insurance) was obtained from patients via a self-administered paper questionnaire. Patients with reading or writing difficulties were assisted by the assistant in completing the questionnaire. Clinical data (comorbidities, duration of type 2 diabetes, cognitive screening test), behavioral data (smoking, alcohol consumption, self-care behavior, physical activity, dietary assessment, and sleep quality), and psycho-social data (depression screening and DRD) were obtained via questionnaire and assessment tool, interviewed by the assistant. If any abnormalities were detected during the data collection, the assistant would promptly notify the attending physician. Otherwise, patients would continue their routine examinations and treatment by physician. Afterward, another trained assistant collected clinical data (weight, height, waist circumference, blood pressure, FBS, HbA1C, and diabetes treatment modalities) from the electronic medical records of that day and checked to ensure completeness, consistency, and reliability. The author had access to information that could identify individual participants during data collection.” (In revised manuscript without track change, line 169-189)

Line 127 – For data extracted from medical records, please state which values were taken. Was data extracted on the same day as patient interview? Was data from upon diagnosis, or the latest value or otherwise? Please state whether extraction was done uniformly across study population. Was the person who the extracted data from medical records also the same person who conducted the interview? Was there any counterchecking to prevent errors in transcribing

- **Response**:
  - The data extracted from medical records were gathered on the same day as the patient interview by another trained assistant and were relevant to the interview date. Data extraction was done uniformly across study population.
  - we describe in data collection part as follow: “Afterward, another assistant collected clinical data (weight, height, waist circumference, blood pressure, FBS, HbA1C, and diabetes treatment modalities) from the electronic medical records of that day and checked to ensure completeness, consistency, and reliability. The author had access to information that could identify individual participants during data collection.” (In revised manuscript without track chang, line 184-189)

Line 128 – As HbA1c is the most important outcome data in this study, describe in detail how HbA1c level is measured and recorded into the medical file (manual? electronic?)

- **Response**:
  - In our OPD, we schedule routine appointments for diabetes patients every 3-4 months for routine check-up, which include blood tests (FBS, HbA1C) and treatment. HbA1C level is measured by hospital laboratory and recorded in electronic medical file.
  - The patients already had an order for HbA1C, based on our inclusion criteria: “Inclusion criteria were: 1) known cases of type 2 diabetic patients with scheduled appointments and HbA1C tests during the study period, 2) age at least 18 years old 3) be able to answer the questionnaire and agree to participate voluntarily.” (In revised manuscript without track change, line 113-116)

Line 153 – Depression is a medical diagnosis. Please state whether depression is physician-diagnosed depression or based on obtaining a score in a questionnaire. Was the questionnaire administrator for 9Q trained or qualified to conduct the questionnaire (i.e. must is be a doctor with training in psychiatry)?

- **Response**: Thank you for your recommendation. I. our study, we used the 9Q questionnaire for screening depression. The assistant who conducted the questionnaire interviews was a nurse who had been trained before conduct data collection, as follow “Clinical data (comorbidities, duration of type 2 diabetes, cognitive screening test), behavioral data (smoking, alcohol consumption, self-care behavior, physical activity, dietary assessment, and sleep quality), and psycho-social data (depression screening and DRD) were obtained via questionnaire and assessment tool, interviewed by the assistant. ” (as described in data collection part in revised manuscript without track change, line 178-182)

Line 187 – Please also state the age range, to better describe the population demographics.

- **Response**: we add age range in result part as follow
  - “The mean age was 66.2 ± 7.3 years, ranging from 44 to 83 years old, with female predominance (52%).” (In revised manuscript without track change, line 224)

Line 189 – Please state the formula for calculation of prevalence values (uncontrolled DM / DRD/ etc.) in your study.

- **Response**:
  - Prevalence of poor glycemic control was calculated by dividing number of poor glycemic control by total patients included (32/127) (In revised manuscript without track change, line 229-230)
  - Prevalence of DRD was calculated by dividing number of patients with DRD by total patients included (25/127) (In revised manuscript without track change, line 248-249)

Table 1 & Table 2

- For occupation, please change “Job / No job” to “In employment/Working/Unemployed/Not working” etc.

- **Response**: we edit as suggested: “In employment/working” and “Unemployed/not working”

- For education level, was the p-value reported twice?

- **Response**: we edit as suggested

- Please clarify whether Married, living with family is the same as Married (as opposed to Single/not married)

- **Response:** yes, “Married, living with family” is the same as married (as opposed to Single, Separated, divorced, widow)

- For diabetes treatment, explain how p-value was derived. Is it a crosstabulation of all treatment or by each treatment type? Please justify whether crosstabulation by each treatment type is appropriate.

- **Response:**
  - For diabetes treatment, the association with poor glycemic control was calculated by crosstabulation by each treatment type to evaluated effect of each medication type.
  - Some patients had more than 1 types of medication. Thus, we evaluate the association of using more than 1 types of medication and poor glycemic control, which found association only in univariable analysis.

- Please denote tests used for deriving p-values. Please confirm the test used for analyzing income, duration of DM and physical activity.

- **Response:** Follow your recommendation, we add test used in the bottom of each table.

Line 215 and 235 – Please consider reporting the regression model significance and fit.

- **Response to** Line 215**:** we used multivariable logistic regression analysis to evaluate association between potential related factors and poor glycemic control.
  - **“**Table 3 presents multivariable logistic regression analysis of association between poor glycemic control type 2 diabetes and potential related factors. Factors significantly associated with poor glycemic control type 2 diabetes were age under 65 years old (p-value=0.001), obesity (p-value=0.041), and being diabetic patient with distress (p-value<0.001).” (In revised manuscript without track change, line 262-265)
- **Response to** Line 235**:** we used multivariable logistic regression analysis to evaluate association between subcomponent and poor glycemic control.
  - “Table 5 presented multivariable logistic regression analysis of association between poor glycemic control type 2 diabetes and DDS-17 dimensions. Emotional distress (p-value=0.006), regimen-related distress (p-value=0.003), and diabetes-related interpersonal distress (p-value=0.015) were associated with uncontrolled type 2 diabetes after adjusted for age, sex, obesity, using sulfonylureas, using two or more types of diabetes medication, depression, and poor sleep quality. (Table 5)” (In revised manuscript without track change, line 302-307)

Line 239 – Please state how adjustment was carried out

- **Response:** We described in statistical analysis section: “multivariable logistic regression analysis was used to evaluate the association between DRD subcomponents and poor glycemic control, adjusting for sex and other factors previously identified as significant in the univariable analysis.” (In revised manuscript without track change, line 209-211)

Discussion

Please limit stating the results multiple times as they were already stated in the results section in paragraph and also in the tables.

- **Response:** Thank you for your suggestion. We re-write discussion with limit stating the results multiple times as follow: “The purpose of this study was to investigate 1) the prevalence and associated factors of poor glycemic controlled type 2 diabetes, and 2) the prevalence of diabetes-related distress (DRD) and association of its subcomponents and poor glycemic control in Maharaj Nakorn Chiang Mai Hospital, Chiang Mai, Thailand. The prevalence of poorly controlled type 2 diabetes in our study was 29.1%, which was lower than in the previous studies, such as 47.3% in Brazil [5], 50.2% in Japan [6], and 84.3% in Uganda [10].” (In revised manuscript without track change, line 315-327)

The discussion was written in a particular format: “The following factors (x,y,z) was associated with uncontrolled type 2 diabetes mellitus, this was consistent with other studies (A,B,C…)”. Although not incorrect, however, the discussion can be improved by using different variations of the format.

- **Response:** we edit as suggested and re-write to
  - **“**The second factor, obesity was also found to be associated with poor glycemic control, which was supported by previous studies [12, 13] **”** (In revised manuscript without track change, line 335-337)
  - “Multivariable analysis in this study revealed a significant association between being diabetic patients with distress and poor glycemic control. This finding was in conformity with the results of previous studies conducted in Japan [20], Saudi Arabia [19], and the United States [41].” (In revised manuscript without track change, line 348-351)

Results were referenced with other supporting studies. However, were there any inconsistent results or opposing studies? Please elaborate on why your results were unique to your community.

- **Response:** We add the result which inconsistent to previous study as follow. In this study we found that medical adherence was not significant associated with poor glycemic control as in discussion: “In addition to the factors previously discussed, previous studies found an association between medical adherence [46], depression [18], and poor sleep quality [17] and poor glycemic control. However, this study found inconsistent results. Medical adherence was not associated with poor glycemic control in this study, possibly due to high adherence among most patients. Additionally, depression and sleep quality showed only potential but not significant association with poor glycemic control, possibly due to the small sample size. Studies with larger sample sizes could provide clearer and more comprehensive findings.” (In revised manuscript without track change, line 378-384)

- Please discuss the effect of confounders on the results of your study. For example, medication adherence plays an important role in the control of glucose levels, was medication adherence assessed? Similarly, were medication doses optimised and were the patients treated with appropriate medications according to the guidelines i.e. monotherapy, combination OAD, intensive insulin. If these issues are not addressed or controlled for – it can be said that that failure to achieve HbA1c targets may also come from medication under-optimisation and medication non-compliance. How about being on concurrent medications that increases blood sugar levels such as long-term corticosteroids?

- **Response:**
  - For medication adherence
    - We had evaluated medical adherence by questionnaire, which was subcomponent of self-care behavior questionnaire, as describes in method section: “Self-care behavior was assessed using a questionnaire developed by Siangdung S. (Cronbach’s $\alpha$=0.70) [29]. This questionnaire consists of 20 questions covering 5 behaviors including eating, medical adherence, exercise, dealing with stress and continuing of treatment. Each question is rated on a scale of 1 (not performing), 2 (perform occasionally), and 3 (perform regularly). The mean score of behavior was categorized into 3 groups of self-care: low (1.00-1.66), medium (1.67-2.33) and high (2.34-3.00).” (In revised manuscript without tack change, line 139-145). The questionnaire was interview by the trained nurse.
    - In this study we found that most of patient has high level of medical adherence and there was no significant association between medical adherence and poor glycemic control as describe in result part:
      - “Regarding medical adherence, a subcomponent of self-care behavior. There were 112 patients receiving medical treatment, most of patients (99.1%) exhibited a high level of adherence (110/112).” (In revised manuscript without track change, line 244-246)
      - “In terms of behavioral factors, there were no significant differences in self-care behavior, medical adherence, physical activity, dietary intake, smoking, and alcohol consumption between the two groups.” (In revised manuscript without track change, line 251-253)
  - This study did not interfere with patient treatment. The patients received treatment according to the guidelines provided by the OPD's physicians, as described in the method section. “The patients received treatment based on routine guidelines, which include both medication with lifestyle modification and lifestyle modifications only.” (In revised manuscript without track change, line 111-113)
  - None of patient in this study used long-term corticosteroids. (In revised manuscript without track change, line 246-247)
