# Supplementary material for: Prevalence and the association between clinical factors and Diabetes-Related Distress (DRD) with poor glycemic control in patients with type 2 diabetes: A Northern Thai cross-sectional study
Source: PLoS One. 2023 Nov 27;18(11):e0294810. doi: 10.1371/journal.pone.0294810 (PMC10681199; doi:10.1371/journal.pone.0294810)
Supplement: Attachment — Submitted filename: response to reviewer2.docx [file pone.0294810.s004.docx]

Reviewers’ comment to the manuscript [PONE-D-23-07649]

entitled “Prevalence and associated factors of poor glycemic control type 2 diabetes: a Northern Thai cross-sectional study.”

Dear Editor and reviewers,

We would like to thank you for the opportunity to revise our manuscript to be qualified for publication PLOS ONE. We have revised and modified some parts of our manuscript as addressed in response to reviewers’ comments. We hope that our responses and revisions would substantially improve the quality of our manuscript and would be qualified for publication in the journal. If there were any further questions or minor points to be addressed or elaborated, please let us know. We would be more than eager to make any further revision.

Reviewer #2:

Title:

- Consider adding DRD in the title to reflect the large portion of research and discussion on DRD in the study and manuscript.

**Response:** Thank you for your comment. As the objectives of this study were to evaluate 1) the prevalence and associated factors of poor glycemic control type 2 diabetes, and 2) the prevalence of DRD and the association of its subcomponents and poor glycemic control type 2 diabetes in Maharaj Nakorn Chiang Mai Hospital, Chiang Mai, Thailand, We edit Manuscript title to “ Prevalence and the Association between Clinical Factors and Diabetes-Related Distress (DRD) with Poor Glycemic Control in Patients with Type 2 Diabetes: A Northern Thai Cross-Sectional Study”.

Introduction: Sufficiently written to provide background for justification of research

Objectives: Clear

Method:

- Line 121: Please change typo in “Estimating and infinite population…” to “Estimating an infinite population…”
  - **Response:** Thank you for your suggestion. We edit manuscript as follow “The

sample size was calculated using the formula for estimating a proportion in an infinite population” [In revised manuscript without track change, line 119-120].

Instrument and data collection:

- This subsection requires re-writing for clarity and transparency. My concerns are 1) English language, grammar and writing and 2) details – There were various data collection and scales being used. Was/were the data collector(s) trained and eligible to use the various tools/scales? Or was specifically trained person required i.e., medical doctor etc.?
  - **Response**: Thank you for your comment.
    - We have checked and corrected grammar as edits in the instrumental and

data collection part. [In revised manuscript without track change, line 138-219]

- All tools/scales were administered by the registered nurse who was trained

to use the tools before data collection. Moreover, data collection was conducted under doctor supervision. In Thailand, the registered nurse can perform tools/scales tests which have been validated in the Thai population under doctor supervision, as described in the following passage:

- “The same registered nurse conducted questionnaire interviews and

performed assessments using standard tools, including comorbidities, duration of type 2 diabetes, smoking, alcohol consumption, cognitive screening (Mini-Cog), self-care behaviors (self-care behavior questionnaire), physical activity (GPAQ), dietary assessments (24-hour dietary recall), sleep quality (PSQI), depression screening (9Q), and DRD assessment (DDS-17).” [In revised manuscript without track change, line 198-202]

- “Our registered nurse received training in the use of these

questionnaires and assessment tools before starting data collection. In Thailand, registered nurses are qualified to conduct screening tests, such as the self-care behavior questionnaire [28], Mini-Cog [27], GPAQ [29], PSQI [31], 9Q [32], and DDS-17 [33], under the supervision of a family physician. If any abnormalities were detected during the data collection, the assistant nurse would promptly notify the attending family physicians.” [In revised manuscript without track change, line 203-208]

- Line 129 – line 131: Please consider combining into 1 sentence and state who (research assistant) and how data was collected (extraction from database etc.)
  - **Response:** Thank you for your suggestion. We edit the data collection part as

follows:

- Demographic data were collected through a self-administered paper

questionnaire, as describe in the following passage: “Subsequently, demographic data were collected through a self-administered paper-based questionnaire, which included information such as age, sex, occupation, personal monthly income, education, marital status, and health insurance.” [In revised manuscript without track change, line 193-195].

- Data, including comorbidities, duration of being type 2 diabetes smoking,

and alcohol consumption were collected through interviews conducted by a registered nurse trained as a data collection assistant, as described in the following passage: “The same registered nurse conducted questionnaire interviews and performed assessments using standard tools, including comorbidities, duration of type 2 diabetes, smoking, alcohol consumption, …” [In revised manuscript without track change, line 198-200].

- Line 132 – State who did the cognitive screening (state the qualification, doctor? Nurse? Bachelor or graduate student?) and whether that person is eligible to do the screening.
- **Response:**
- In our study, cognitive screening (Mini-Cog) was conducted by the

registered nurse who had been trained to use the test before data collection, as described in the data collection part [In revised manuscript without track change, line 200].

- In Thailand, registered nurses can conduct screening tests (Mini-Cog),

which have been validated in the Thai population under doctor supervision, as described in the following passage: “Our registered nurse received training in the use of these questionnaires and assessment tools before starting data collection. In Thailand, registered nurses are qualified to conduct screening tests, such as the self-care behavior questionnaire [28], Mini-Cog [27], GPAQ [29], PSQI [31], 9Q [32], and DDS-17 [33], under the supervision of a family physician. If any abnormalities were detected during the data collection, the assistant nurse would promptly notify the attending family physicians. [In revised manuscript without track change, line 203-208]

- Line 139 – Is this “questionnaire” a patient-reported questionnaire or an interviewer-guided questionnaire? Please state in manuscript. Please state whether the person who the extracted data from medical records also the same person who conducted the interview? Was there any counterchecking to prevent errors in transcribing?
  - **Response:**
    - The registered nurse conducted questionnaires interview, as described in

the following passage: “The same registered nurse conducted questionnaire interviews and performed assessments using standard tools, including comorbidities, duration of type 2 diabetes, smoking, alcohol consumption, cognitive screening (Mini-Cog), self-care behaviors (self-care behavior questionnaire), physical activity (GPAQ), dietary assessments (24-hour dietary recall), sleep quality (PSQI), depression screening (9Q), and DRD assessment (DDS-17). Our registered nurse received training in the use of these questionnaires and assessment tools before starting data collection.” [In revised manuscript without track change, line 198-204]

- The person who extracted data from the medical records was a research

data collection assistant (the first research assistant physician and not the same person who conducted the interviews), as described in the following passage: “Afterward, a research data collection assistant, who was not involved in the collection of questionnaire and patients’ treatment, retrieved data including weight, height, waist circumference, blood pressure, FBS, HbA1c, and diabetes treatment modalities from the electronic medical records of that day.” [In revised manuscript without track change, line 211-214]

- We have another research assistant (the second research assistant

physician) who countercheck data, as described in the following passage: “Another research assistant was assigned to double-check the data for completeness, consistency, reliability, and to prevent transcription errors” [In revised manuscript without track change, line 214-215]

- Line 157 –Was the questionnaire administrator for 9Q trained or qualified to conduct the questionnaire (i.e. must be a doctor with training in psychiatry)?
  - **Response:**
    - Thank you for your comment. In our study, 9Q was conducted by the

registered nurse who had been trained to administer the test before data collection, data collection was conducted under doctor supervision. In Thailand, registered nurses can conduct 9Q tests, as described in the following passage:

- “The same registered nurse conducted questionnaire interviews and

performed assessments using standard tools, including comorbidities, duration of type 2 diabetes, smoking, alcohol consumption, cognitive screening (Mini-Cog), self-care behaviors (self-care behavior questionnaire), physical activity (GPAQ), dietary assessments (24-hour dietary recall), sleep quality (PSQI), depression screening (9Q), and DRD assessment (DDS-17).” [In revised manuscript without track change, line 198-202]

- “Our registered nurse received training in the use of these

questionnaires and assessment tools before starting data collection. In Thailand, registered nurses are qualified to conduct screening tests, such as the self-care behavior questionnaire [28], Mini-Cog [27], GPAQ [29], PSQI [31], 9Q [32], and DDS-17 [33], under the supervision of a family physician.” [In revised manuscript without track change, line 203-207]

- Line 186 – As HbA1c is the most important outcome data in this study, describe in detail how HbA1c level is measured (etc. whole blood? Fasting state? Point of care?)
  - **Response:** Thank you for your suggestion. We have added information about

HbA1c collection in the research instrument and measurements, as described in the following passage: “Moreover, data including weight, height, waist circumference, blood pressure, fasting blood sugar (FBS), glycated hemoglobin (HbA1c), and diabetes treatment modalities were obtained from the electronic medical record. For FBS and HbA1c, whole blood specimens were collected by licensed medical technicians on the same day as the data collection. Patients fasted (nothing per oral, NPO) for 12 hours prior to blood collection. All specimens were properly stored and analyzed by our central laboratory on the same day. The results were then recorded in the electronic medical record.” [In revised manuscript without track change, line 174-180]

Results:

- Table 1 & 2: Please clarify the use of Fisher’s exact test for the comparison of median values between groups.
  - **Response:** Thank you for your comment. Actually, we used the Mann-Whitney U

test for comparing median values between groups (there was a typographical error in the previous manuscript). However, we have made the necessary edits in the revised manuscript for both table 1 and table 2.

- Table 3 & 5 – Please consider reporting the regression model significance and fit.
  - **Response:** Thank you for your comment on this point. However, the presentation

of regression model significance and model fit parameters is not generally required in the context of exploratory factor research, as is the case with our study. Model fit parameters, such as goodness-of-fit tests or calibration, are specific requirements for studies that develop and present clinical prediction/regression models.

Discussion

- Line 322 – Please check and correct the unit; whether mg/dL or mmol/L.
  - **Response:** Thank you for your comment. We have checked the unit of HbA1c(%)

and edited it as described in the following passage: “The difference in prevalence might be attributed to variations in HbA1c target goals. Some countries adopt an HbA1c goal of less than **7%** [6, 10], while others follow the HbA1c goal outlined in the ADA guideline for 2021[5], which allows for greater flexibility, especially for older patients.” [In revised manuscript without track change, line 341-344]

- Professional proofreading is recommended to elevate writing quality of discussion section.
  - **Response:** Thank you for your suggestion. We obtained professional proofreading

to improve the quality of our discussion section. [In revised manuscript without track change, line 336-425]

Conclusions
Conclusions were well-written.

General comments
Majority of the references are recent publications (published within 10 years).
